# Assessing Associations Between Environmental, Sleep, and Physical Activity Factors and Metabolic Syndrome Risk: Protocol for the FEASible Study

**DOI:** 10.2196/82034

**Published:** 2026-01-06

**Authors:** Tania Ramos-Santiago, Elise Huglo, Yanrong Li, Christian Corral, Congyu Wu, Kerry Kinney, Darla Castelli, Andreana Haley

**Affiliations:** 1 Department of Psychology University of Texas at Austin Austin, TX United States; 2 Maseeh Department of Civil, Architectural, and Environmental Engineering University of Texas at Austin Austin, TX United States; 3 Department of Systems Science and Industrial Engineering Binghamton University Greater Binghamton, NY United States; 4 Department of Physical Therapy, Movement, and Rehabilitation Sciences Northeastern University Boston, MA United States

**Keywords:** indoor air quality, Latina women’s health, metabolic health, metabolic syndrome, MetS, physical activity, wearable sensors

## Abstract

**Background:**

FEASible is a cross-sectional observational study that explores women’s daily living patterns through wearable devices and home environment sensors to validate the use of physical activity and indoor air quality data as indicators of risk for metabolic syndrome (MetS) and cardiovascular disease.

**Objective:**

Leveraging transdisciplinary expertise, we implement a low-cost, dense sampling approach among 800 adult women, 60% Hispanic or Latina, with a subgroup of 225 participants opting for neuroimaging to assess MetS-related brain vulnerability.

**Methods:**

Participants residing in Central Texas use a smartwatch and a custom-built air quality sensor to monitor their activities and environment for two weeks. Other variables, such as social determinants of health, medical history, and lifestyle, are reported through surveys. During their initial visit, we gather blood pressure measurements, body composition, and lipid profile information.

**Results:**

As of August 2025, a total of 805 participants have completed the eligibility survey, of whom 204 have completed 2 weeks of sensor data collection, showing a diverse participant pool comprising 60% (210/348) Hispanic or Latinas, 39% (135/348) non-Hispanic or Latinas, and 1% (3/348) unknown. Participants are aged 18-40 years, with an average age of 27 years (SD 6 years). The study was funded in May 2023 (National Heart, Lung, and Blood Institute; grant R01HL168374), and data collection began in October 2023, with a projected completion date of May 2028. Ongoing analyses and publication of early cohorts are expected to occur throughout 2026 and 2027, with final analyses and dissemination of results anticipated by the end of the funding period in 2028.

**Conclusions:**

We have established a feasible pipeline to collect data that will help yield valuable insights into MetS risk factors among Latina women, including brain scan magnetic resonance imaging data and environmental exposure measurements. This paper aims to provide a rationale for procedures and case examples from the first year of data collection leading to health risk modeling, thus informing future interventions on MetS, heart disease, and all-cause mortality among Latina women.

**International Registered Report Identifier (IRRID):**

DERR1-10.2196/82034

## Introduction

Nearly 1 in 5 people in 2022 identifies as Hispanic or Latino, making this population segment one of the most significant and fastest-growing racial or ethnic minorities in the United States [1]. Texas has been categorized as having one of the nation’s largest Hispanic and Latino state populations, accounting for 12.1 million people [2]. Statistics show that Hispanic and Latino communities, a substantial proportion of the population in Texas, face a unique set of health challenges. Among these, cardiovascular diseases (CVDs) are one of the leading causes of death in Hispanic and Latina women in the United States [3,4]. CVD is a broad term covering various heart and blood vessel diseases. Metabolic syndrome (MetS) is a cluster of risk factors specific to CVDs that includes abdominal obesity, elevated fasting glucose, elevated triglycerides, low-density lipoprotein cholesterol, and high blood pressure. MetS has been independently associated with and is considered a precursor to CVDs and mortality [5,6]. While Hispanic and Latina women generally exhibit a higher prevalence of most MetS risk factors compared with non-Hispanic White women, our understanding of what environmental or behavioral factors contribute to these disparities remains limited to prescribed medications, self-report surveys, and risk ratios [7-9]. Social determinants, such as socioeconomic status, access to health care, and lifestyle behaviors, also contribute to the elevated risk of heart disease in this population [10]. Considering that Hispanic and Latino communities are growing fast in the United States, the higher prevalence of MetS in women, and the trends of health disparities after the global pandemic, a critical missing component that needs to be further understood is the correlation of daily behavioral activities with MetS risk.

There is an increasing focus on studying health, environmental, and behavioral patterns through cost-effective and widely available wearable sensors to understand and improve public health. The success of this endeavor is critically dependent on accurate models reflecting unique daily lifestyles, ultimately guiding potential interventions to promote health equity through behavioral interventions. Smartwatches and other wearable devices have garnered considerable attention, as they can help track key health indicators, including sleep patterns, heart rate, and physical activity. Indeed, poor physical activity and sleep time have been consistently associated with an increased risk for MetS in women [11-13]. However, evaluating Hispanic and Latina women’s unique environmental exposures and cardiovascular risks is a significant gap in the MetS risk framework.

A second set of essential parameters to monitor environmental health risks is indoor air pollutants and other relevant measures of the indoor environment, such as temperature and humidity. Indeed, adults spend 90% of their time indoors, but this aspect is not generally evaluated as rigorously as it should be [14-16]. Specific sensing technologies now make this possible at a lower cost. For example, particulate matter ≤2.5 µm in diameter (PM_2.5_) is a widely studied outdoor air pollutant in urban areas. It has been found to directly affect the respiratory system, hypertension, physical functioning, and cognitive performance [17-19]. Moreover, chemical exposure affects cognitive function; for example, carbon dioxide (CO_2_) is predominantly present in indoor environments and has also been linked to reduced cognitive function and productivity [20]. The development of portable environmental sensing devices offers a promising method for monitoring indoor air quality. However, it is imperative to validate the reliability and accuracy of these low-cost wearables and custom-made air quality sensing devices to better measure attributes of daily living and quantify MetS risk. To establish the utility of these devices in personalized interventions, a thorough calibration and validation of the developed sensing device models are necessary to capture relevant, ecologically valid data in the home environment.

The FEASible study purpose is divided into 3 specific aims to validate a pipeline of low-cost objective measurements and interpret MetS risk and MetS-related brain vulnerability networks. The primary objective is to identify which modifiable peripheral cardiometabolic biomarkers are associated with MetS-related brain vulnerability in Latina women, serving as a ground-truth standard. The hypothesis is that MetS, peripheral inflammation, white and gray matter volume, white matter hyperintensities, and measures of cerebral metabolism will form a network of MetS risk and brain vulnerability. The second aim is to validate the use of low-cost sensors that capture multiple environmental measures inside and around the home to assess exposures that may affect sleep and physical activity. We will use this information to identify potential risk factors for MetS and MetS-related brain vulnerability. The hypothesis states that properly validated, low-cost sensors can capture sleeping habits and home environmental conditions that contribute to more disordered sleep and less participation in physical activity, which in turn mediates MetS outcomes. The third aim is to determine the feasibility and association of mobile sensor measurements of daily behavior, activity, sleep, and environmental attributes with MetS and MetS-related brain vulnerability. The first hypothesis posits that features of daily living detected by mobile sensors will more accurately correlate with MetS risk and MetS-related brain vulnerabilities than participant baseline information. The second hypothesis is that environmental exposures detected by validated, low-cost monitoring devices will mediate the relationships between mobile and wearable sensor measurements of daily behavior and MetS risk and MetS-related brain vulnerabilities. Validating low-cost and objective measurements for MetS risk will aid in preventing MetS development, heart diseases, and all-cause mortality. Given the limited research on Latina women, identifying reliable and accurate customizable devices that target individual behaviors and environments is crucial for designing prevention efforts with greater precision.

## Methods

### Study Design

**Figure 1:**
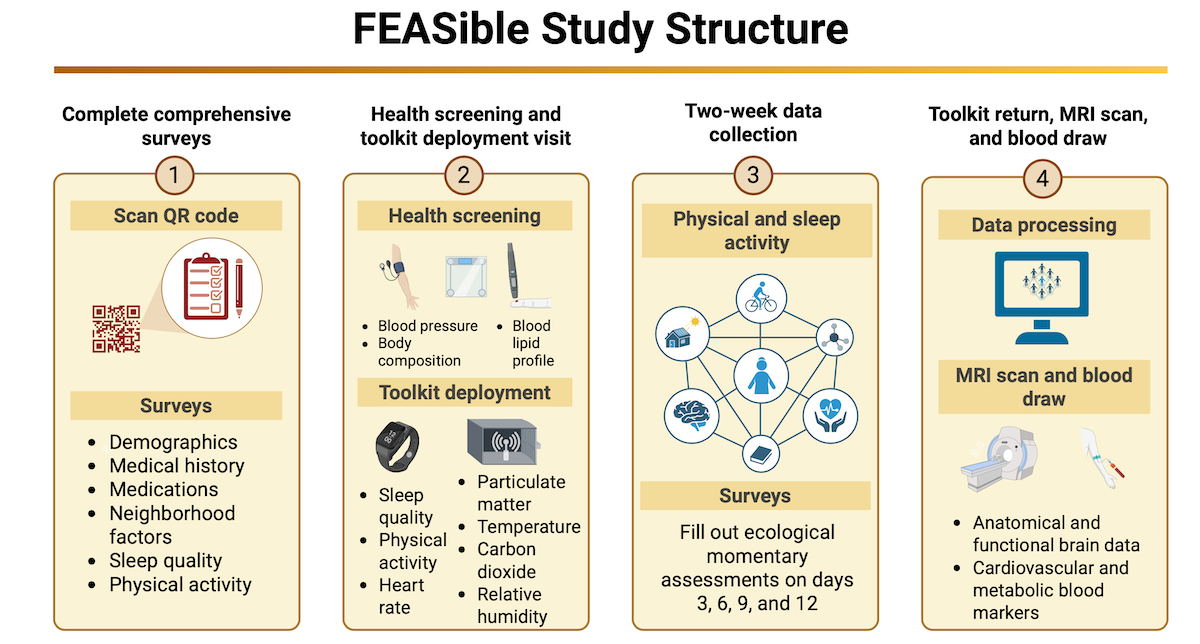
The FEASible study structure. Participants are asked to complete comprehensive surveys, followed by performing a health screening and a toolkit deployment visit where they get the smartwatch and the home environment sensor kit. The participants complete the 2-week data collection, and after they return the toolkit, they might opt to participate in the magnetic resonance imaging (MRI) scan and blood draw. Created with BioRender by Ramos et al [21], reproduced using license obtained by authors.

FEASible is a cross-sectional study conducted at an academic clinical neuroscience research laboratory. Each month, groups of 25-40 adult women are onboarded in the study. The study is active from October 2023 until May 2028. Figure 1 summarizes the study structure. First, the participant completes an eligibility survey through REDCap (Research Electronic Data Capture; Vanderbilt University), followed by informed consent and comprehensive surveys. Then, the participant visits the laboratory to perform the health screening, set up the wearable device, and deploy the home environment sensor kit. Participation is required for a minimum of 14 to 30 days, during which dense sensor capture of daily living is conducted. After completing the 2-week data collection, the participant returns to the university to return the toolkit. The participant can schedule a subsequent visit for the in-laboratory blood draw and magnetic resonance imaging (MRI) scanning if eligible.

The research participant population of interest consists of adult women, with 60% (210/348) identifying as Latinas. The total sample size comprises 800 participants, with a subgroup of 225 participants opting to participate in neuroimaging. The inclusion criteria are (1) aged 18-40 years during data collection, (2) having no advanced health conditions or illness (eg, brain tumor, psychotic disorder, or history of neurological conditions), (3) not currently pregnant, (4) absence of a physical disability that inhibits mobility, and (5) living in Central Texas (2-hour radius of Austin, Texas). For the neuroimaging subgroup, the additional inclusion criteria are (1) no MRI contraindications, (2) no diagnosed medical diseases or psychological disorders, and (3) no history of head injury with loss of consciousness greater than 5 minutes. All study participants are compensated for completing the comprehensive surveys, participating in the 2-week data collection, and undergoing the MRI scan.

### Sample Size and Power Analysis

To demonstrate statistical power, we conducted a simulation analysis for the anticipated experiments to test our hypotheses, aiming to determine the extent to which mobile-sensed behavioral features can correlate high versus low levels of risk for MetS. Datasets were simulated as random draws from a multivariate distribution with a ground-truth covariance based on sensing data collected from the 1012 participants for a 21-day study period during the Fall 2018 deployment of our previous UT1000 study [16,22,23]. We consider a 24-hour day to consist of four 6-hour epochs, namely morning (6-12 hours), afternoon (12-18 hours), evening (18-24 hours), and night (24-6 hours).

### Recruitment Strategies and Screening

The recruitment strategies are flyers, business cards, word of mouth, Latina-focused Facebook groups, and Latino community events. To ensure a representative sample, we establish research partnerships with the community, local groups, and nonprofit organizations, while collaborating to offer health and environmental resources, including talks and educational materials. Our primary recruitment strategy is word of mouth, posting flyers in targeted areas, and sending text messages via partnerships with the Special Supplemental Nutrition Program for Women, Infants, and Children partnership. First, participants are screened through a REDCap eligibility survey. REDCap is a secure web application designed to support data capture for research studies. If the participant meets the inclusion criteria, they are invited to complete an informed consent form and a demographics and contact information survey to schedule the initial visit. All materials are available both in English and Spanish.

### Instruments and Surveys

There are two types of survey instruments: (1) screening and characterization, and (2) data collection. The screening instruments are used to determine eligibility and confirm living arrangements (Table 1), while the survey instrument provides valid, reliable self-report data (Table 2). All instruments are hosted on REDCap and distributed by email, through QR code, or by text message.

**Table 1:** Screening and characterization.

Survey name	Measured constructs and validity
Eligibility	A total of 10 response (yes or no) questions listing the inclusion criteria. Automatically scored. If eligible, the participant is sent to the study dashboard to read the study overview and consent to be contacted by the research team.
Consent	Participants are provided with an overview of the study and confirm their willingness to participate. Consent is again confirmed through scheduling meetings and during the on-site visit.
Demographics	Information about themselves (eg, age) and their living situation (eg, how many people live with them).

**Table 2:** Data collection.

Survey name	Measured constructs and validity
Medical history (laboratory visit 1)	A total of 25 forced response (yes or no) questions listing general medical problems, conditions, or behaviors. Five questions had follow-up prompts (eg, smoking, when did you quit smoking?).
Current medications (laboratory visit 1)	A total of 20 forced response (yes or no) questions with a follow-up prompt and an open text box when there was a “yes” reply.
Neighborhood Factors Questionnaire (laboratory visit 1)	A total of 7 subscales of walkability, aesthetic quality, safety, violence, availability of healthy foods, social cohesion, and activities with neighbors as factors associated with physical activity [24].
Groningen Sleep Quality Scale (laboratory visit 1)	A total of 15 true or false questions about sleep quality (eg, I slept well last night) [25].
Mini MASQ^a^ (laboratory visit 1)	A total of 26 Likert scale questions about perceptions [26,27].
IPAQ^b^ (laboratory visit 1)	A total of 27 questions about physical activity duration and intensity enable the calculation of the metabolic equivalent of task-minutes as a measure of physical activity volume [28].
Everyday Discrimination Scale–Everyday Experiences Source (laboratory visit 1)	This brief survey consists of 10 Likert scale questions that identify the frequency of perceived discrimination (eg, never to almost every day) as an indicator of stress and an increased risk for MetS^c^. The following prompt identified the potential reason they thought this occurred (gender, body image, etc) [29].
Home toolkits	The toolkits contain a commercial Fitbit smartwatch (Fitbit Inc) and a custom-built air quality sensor.
EMA^d^	EMA involves the repeated sampling of the participants’ behaviors and experiences in real-time in natural environments. EMAs aim to minimize recall bias and maximize ecological validity. Once the toolkits are deployed, EMAs are sent via mobile phones to confirm study compliance and to document whether any unusual circumstances occurred. Participants receive a comprehensive set of surveys during the first days of data collection and an EMA on Days 3, 6, 9, and 12 for a total of 4 EMAs [30].

^a^MASQ: Mood and Anxiety Symptoms Questionnaire.

^b^IPAQ: International Physical Activity Questionnaire.

^c^MetS: metabolic syndrome.

^d^EMA: Ecological Momentary Assessment.

### Procedures

Once consent is confirmed, the participants are scheduled for their first in-person health screening. Before arriving, they are asked to complete the survey data collection through REDCap (Table 2). Participants can also volunteer to be screened for eligibility for the MRI phase of the study, which is a second laboratory visit.

#### Laboratory Visit 1: Health Screening and Toolkit Deployment

During the health screening, we measure anthropometric parameters such as body composition, blood pressure, lipids, and glucose levels. Height, weight, BMI, fat mass percentage, waist circumference, and visceral adipose tissue are measured using a Seca body composition scale (seca mBCA 555/554; seca GmbH & Co KG) through bioelectric impedance. The participant is asked to stand on the platform barefoot for around a minute. For blood pressure measurements, an Omron 5 series upper arm pressure monitor (VP-2000; Omron Healthcare) is used to measure brachial systolic, diastolic, and pulse. It is assessed in the seated position after a 10-minute rest. Three measurements are taken, with a 3-minute interval between each measurement. Additionally, a blood screening using a Cholestech LDX (Alere) is conducted to measure participants’ blood lipids and fasting glucose levels. The participant must have fasted for 8 hours before performing the finger prick. A finger prick is done using a lancet on the side of the center finger in the nondominant hand. The sample is collected through a capillary tube of 40 µL (Ref 52193, Abbott) and dispensed with a capillary plunger (Ref 10311, Abbott) into a Cholestech LDX Lipid Profile Glucose Cassette (Ref 97991, Abbott). Values obtained by the Cholestech LDX include blood concentrations of total cholesterol, triglycerides, high-density lipoprotein cholesterol, low-density lipoprotein cholesterol, and glucose.

#### Data Return

**Figure 2:**
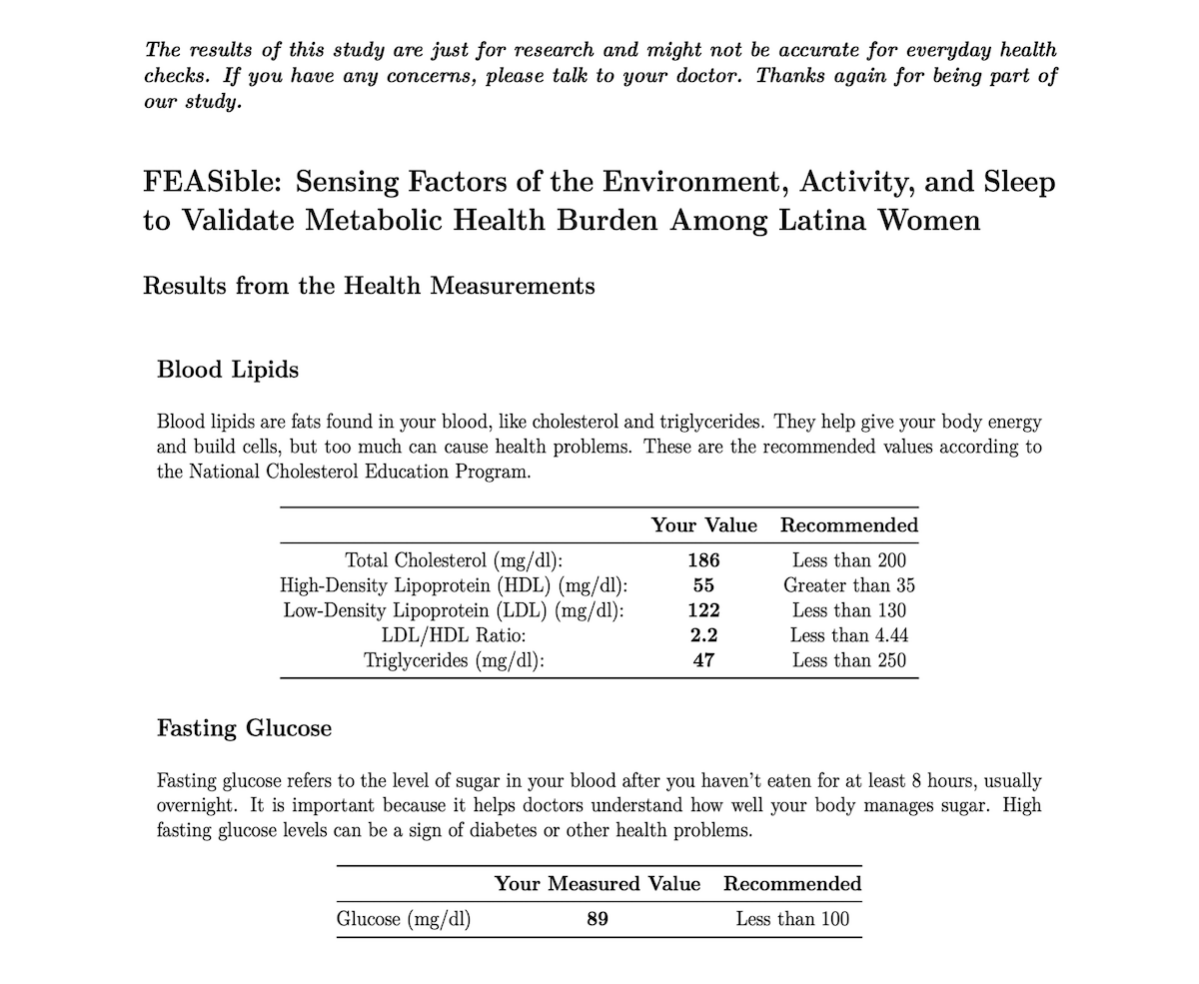
Sample health screening data return. Every participant receives a report with their health measurement results.

In line with best practices in health research and to strengthen rapport with our participants, we provide a concise report of the health screening information [31]. We used the publicly licensed R Markdown (RStudio, PBC) to create personalized, automated reports (Figure 2) [32,33].

The reports are generated using an R code that references data from the REDCap report function and creates a ready-to-send document for each participant. The report is designed with ease of comprehension in mind, written at an eighth-grade reading level, and includes tables that provide recommended biomarker ranges. They are used as an informational tool for participants to understand their engagement with our research. Health recommendations are compiled from the National Cholesterol Education Program (National Heart, Lung, and Blood Institute) and are publicly available information. Our participants understand that this information is for research purposes only and not intended for clinical diagnosis. If they request, we provide additional health literacy resources from the American Heart Association regarding MetS. We consider this a key component of our engagement with participants and essential in establishing trust and transparency with our participant community.

#### Toolkit Deployment

**Figure 3:**
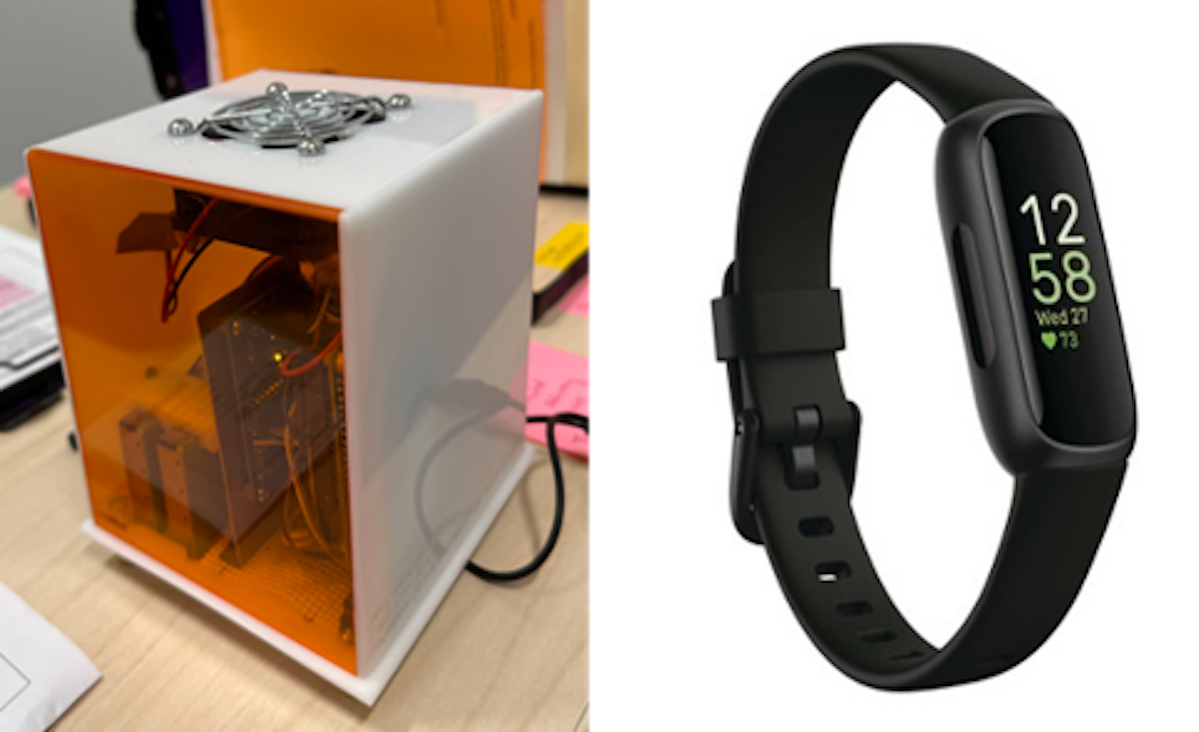
The sensing devices used for the FEASible Study in the home environment sensor kit (left) and Fitbit Inspire 3 (right).

After measuring weight and height as part of the health screening, the participant downloads the Fitbit phone app, where the values are used to accurately measure the calories burned throughout the day, step counts, and heart rate. A commercial Fitbit Inspire 3 (Figure 3) is deployed for use by the participant by entering age, sex, and body weight.

The research assistant provides instructions on how to use the Fitbit, including how to navigate the Fitbit App dashboard, charge and maintain the battery, and verify that the device is collecting data accurately. Additionally, participants can try several Fitbit bands to find the one that suits them best in terms of tightness. Participants can track their daily exercise, calorie intake, distance walking or running, and sleep cycles. Sleep cycles are categorized into 4 stages: awake, light, deep, and rapid eye movement.

The participant is expected to wear the Fitbit continuously for 14 days. Participants are instructed to remove the device while bathing and to charge it for 30 minutes. Since the Fitbit Inspire 3 has excellent battery life, it requires only 1 or 2 charging sessions in 2 weeks. Of the 178 participants completed by August 2024, a total of 148 (83%) wore the Fitbit for at least 14 continuous days (mean 17.85 days). The Fitbit device was configured to capture an energy expenditure reading every minute; thus, it would return 24×60=1440 readings per day if worn the entire day without interruption. We calculated a completeness metric for each participant by dividing the actual number of calorie readings by 1440. Out of the 148 participants who wore the Fitbit for at least 14 continuous days, the mean completeness was 93.45%. These numbers indicate that we have collected at least 2 weeks of continuous Fitbit data.

While Fitbit devices are not considered clinical gold standards, they provide accessible and scalable measures that serve as potential proxy features for modeling health risk patterns. To ensure data reliability, we will apply multiple data cleaning and preprocessing steps. Specifically, days with zero variation in minute-by-minute calorie data will be removed, which likely indicate nonwear periods. For each participant, a completeness metric will be calculated, representing the percentage of nonmissing readings out of the total 1440 minutes per day. Only days with at least 50% valid data will be retained for subsequent analyses. Minor gaps within valid days will then be interpolated to maintain temporal continuity. These procedures will help ensure that wearable data used in modeling reflect actual participant activity and sleep behavior rather than device artifacts or missing data.

For the home environment sensor kit, a custom-built environmental sensor (noncommercial device) (Figure 3) is given to the participant to place in their home at the same time as Fitbit usage. The participant is instructed to plug the home environment sensor kit into an AC outlet and to place the device at dresser height (or equivalent) in the bedroom for 1 week and on a countertop (away from the stove) in the kitchen for the second week.

#### Air Quality Sensors

The home environment sensor kit is an inexpensive, custom-made, real-time sensor and logger developed in the laboratory. It collects continuous temperature, relative humidity, CO_2_, and PM_2.5_ time-series data at 1-minute sampling intervals. Version 2 of the environmental sensor is deployed in the FEASible study. Version 1 previously successfully collected data to evaluate the association between sleep and indoor air quality among university students [34].

The home environment sensor kit comprises 5 sensors (Table 3): one Sensirion SCD-30 for CO_2_, which has integrated temperature and relative humidity sensors as well, two Sensirion SPS-30 for PM_2.5_, one Honeywell HIH-8120-021 to measure relative humidity, and one TTS-1KC3-BZ thermistor for temperature measurement. Every minute, a measurement is logged on a spreadsheet. Low-cost sensors offer several advantages, including small size, mobility, lightweight design, affordability, and ease of use, which enable an increase in spatial and temporal resolution [35]. Meanwhile, measurement uncertainty is more significant, and frequent calibration is needed. Particle sensors are typically provided with manufacturer-installed calibrations that depend upon the manufacturer’s specific calibration condition; information about these conditions is usually unavailable. Individual calibration of each sensor under controlled conditions is necessary to characterize uncertainty in the data and improve cross-device accuracy. The first calibration round of 20 home environment sensor kits was performed in a controlled environmental chamber against research-grade instruments: a TSI APS 3321 aerodynamic particle sizer and a LI-COR LI-7000 CO_2_ analyzer. CO_2_ and PM_2.5_ calibration data were collected simultaneously using Arizona Test Dust (12103-1, A3 Medium Test Dust, Powder Technology Inc, PTI ID 14891M) as a standard for PM_2.5_ calibration through a spike and decay process.

**Table 3:** Home environment sensor kit specifications.

Sensor and type name	Sensing	Range	Characteristics
Sensirion SCD-30 (transmissive NDIR^a^)	CO_2_^b^	400 ppm to 10,000 ppm	Accuracy: ±(30 ppm +3%)
Sensirion SPS-30 (optical, laser scattering)	PM_2.5_^c^	0.3 μm to 2.5 μm	Precision: ±100 #/cm³ for range 0-1000 #/cm³; +10% mv^d^ for range 1000-3000 #/cm³
Honeywell HIH-8120-021 (thermoset-polymer capacitive sensing element)	Humidity	0% to 100% RH^e^	Accuracy: ±2.0% RH
TTS-1KC3-BZ (thermistor with a Maxim MX7705 ADC^f^ thermistor)	Temperature	–40 °C to 125 °C	Tolerance (0 °C to 70 °C): ±0.2 °C

^a^NDIR: nondispersive infrared.

^b^CO₂: carbon dioxide.

^c^PM_2.5_: particulate matter ≤2.5 μm in diameter.

^d^mv: measured value.

^e^RH: relative humidity.

^f^ADC: analog-to-digital converter.

The home environment sensor kit design enables users to verify the validity of the measurements from different sensors. There are 2 particulate matter sensors, 2 temperature sensors, and 2 relative humidity sensors. Therefore, if one drifts unusually and unexpectedly, it can be caught, replaced, and recalibrated before the next deployment. We performed regular calibrations through the first year of deployment.

#### Laboratory Visit 2: MRI and Blood Draw Visit

After the toolkit deployment visit, participants are asked for interest in an additional visit for MRI scanning and blood draw. Those responding positively are checked for the absence of contraindications to MRI over the phone. To minimize distortion, stricter exclusion criteria are applied to any metal implant in proximity to the head, even if the material is MRI-safe. Eligible participants obtain detailed information about the visit procedures and compensation. Once the participant agrees, an appointment is scheduled for the morning, lasting approximately 1.5 hours. A consenting subgroup of 225 women will participate in the neuroimaging component of the study, which includes a comprehensive MRI scan session and an in-laboratory blood draw.

On the day of the MRI scan and blood draw visit, participants are instructed to fast for 8 hours before the blood draw. Plasma and serum blood samples are collected from the antecubital vein through venipuncture and centrifuged at room temperature for 10 minutes at 1300 rpm in a Centrifuge 5810 (Eppendorf AG). However, the plasma tube is centrifuged immediately after collection, and the serum tube is left at room temperature for 30 minutes before centrifugation. After centrifugation, 0.5 mL of the supernatant from plasma and serum tubes is transferred to three 12 × 75-mm tubes and immediately stored at –80 °C for later analysis of inflammatory markers.

After the blood draw, the participant is escorted to the MRI scan. The MRI scans are conducted using a 3-Tesla PRISMA scanner (Siemens Healthineers). Upon arrival, participants undergo a comprehensive MRI safety screening and receive a detailed explanation of the procedures. Participants are asked to remove any personal clothing and accessories and change into medical scrubs to eliminate any potential sources of metal interference. During the scan, participants are instructed to remain as still as possible and keep their eyes open during the pseudo-continuous arterial spin labeling (pcASL) and blood oxygenation level–dependent functional MRI (BOLD fMRI) sequences. They are encouraged to communicate any discomfort or emergencies to the research staff at any time and are informed of their right to terminate the session if needed.

The protocol includes the following sequences: T1-weighted magnetization prepared rapid gradient echo (MPRAGE; Echo Time [TE]=2.01 ms, repetition time [TR]=2000 ms, inversion time [TI]=900 ms; flip angle=8 degrees; voxel dimension=1.0 × 1.0 × 1.0 mm³; field of view [FOV]=256 × 256 mm; total acquisition time [TA]=4:40); T2-weighted 2D fluid-attenuated inversion recovery (FLAIR; TE=91 ms, TR=9000 ms, TI=2500 ms; voxel dimension=0.9 × 0.9 × 3.0 mm³; FOV=220 × 220 mm; TA=4:05); magnetic resonance spectroscopy (MRS) localizer (TE=1.85 ms, TR=1280 ms, TI=725 ms; voxel dimension=0.4 × 0.4 × 1.1 mm³; FOV=224 × 210 mm; TA=2:02); single voxel spectroscopy (SVS) spin echo (SE) spectroscopy, water suppressed (TE=30 ms, TR=4750 ms; voxel dimension=31 × 16 × 16 mm³; bandwidth=1600 Hz; TA=5 seconds to 7 seconds; water suppression=weak water suppression); SVS SE spectroscopy, unsuppressed (TE=30 ms, TR=4750 ms; voxel dimension=31 × 16 × 16 mm³; bandwidth=1600 Hz; TA=5 seconds to 7 seconds; water suppression=RF off); and unsuppressed relaxation-resolved advanced multi–spin-echo chemical shift (RRAMSC) sequences (TE=30 ms to 260 ms, TR=1030 ms to 2150 ms voxel dimension=31 × 16 × 16 mm³; bandwidth=1600 Hz; TA=5 seconds to 7 seconds). The total acquisition time was approximately one hour.

### Ethical Considerations

This study protocol is approved by the University of Texas’s Institutional Review Board (STUDY00003675). To date, 12 Institutional Review Board–approved modifications have been made, primarily to add study personnel, adjust compensation procedures, and clarify protocol details. The informed consent process is obtained in two phases. First, participants complete an eligibility survey; those eligible are prompted to review and electronically sign the informed consent form before proceeding. Second, at the initial laboratory visit, informed consent is reviewed again, and study staff assess comprehension and address any questions. To ensure participant confidentiality, only authorized individuals may access identifiable information for research oversight. However, all participants are assigned a code, not their name. Lastly, participants are compensated by gift cards: US $50 for survey completion, US $200 for contributing sensor data, and an additional US $150 for participants who are eligible and consent to neuroimaging and blood collection. Participants who withdraw early will receive prorated compensation.

## Results

### Overview

Year one data and selected cases are reported as initial evidence of the procedure used in the FEASible research study. Data collection started in 2023 and will continue until 2028. Some variables in this section are reported as aggregate scores, including eligibility, demographics, and survey summary scores, whereas other variables display results from a single case to illustrate the collected data.

**Figure 4:**
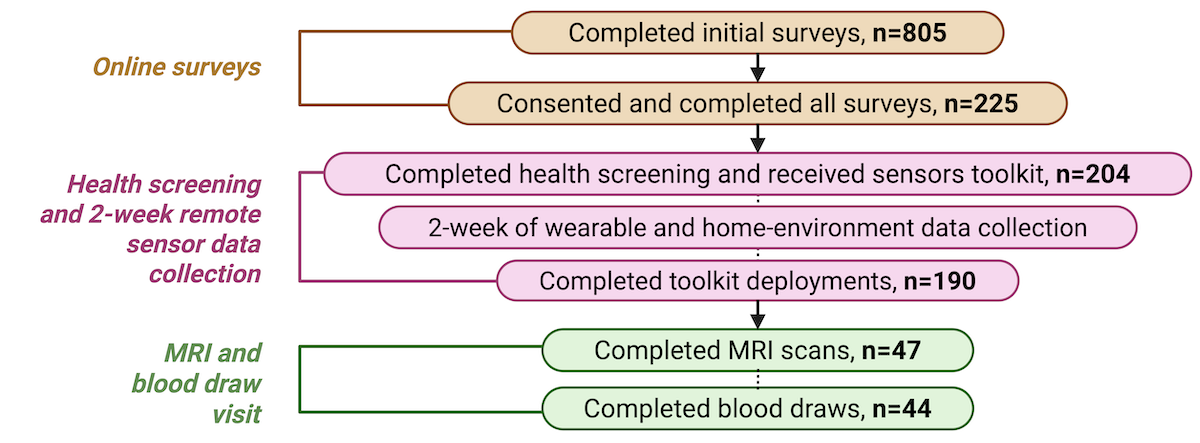
The FEASible study CONSORT (Consolidated Standards of Reporting Trials) diagram. The study is divided into 3 main categories: online surveys, health screening, 2-week remote sensor data collection, and magnetic resonance imaging (MRI) and blood draw visits. The n represents the number of participants. Created with BioRender by Ramos et al [36], reproduced using license obtained by authors.

Out of the total of 805 participants screened for eligibility, a total of 225 consented to participate in the study and complete all surveys (Figure 4). Of these, 204 completed the health screening and received sensor toolkits for collecting wearable and home environment data over the 2 weeks. Lastly, a subset of these participants (n=47 and n=44) underwent MRI scans and blood draws, respectively. Table 4 summarizes demographics collected from the initial eligibility survey; these data reflect potential participants, not those who completed the full study.

**Table 4:** Participant demographics from Year one initial eligibility surveys. Percentages are calculated within each demographic factor. Totals within a factor may not equal totals in other factors due to missing responses or participants selecting multiple categories.

Factors		Frequency, n (%)
**Age (years)**		
	18-25	169 (49)
	26-33	126 (36)
	34-40	52 (15)
**Latina heritage**		
	Yes	210 (60)
	No	135 (39)
	Do not know	3 (1)
**Latina origin**		
	Central American	23 (11)
	Cuban	5 (2)
	Dominican	1 (1)
	Mexican	138 (63)
	Puerto Rican	13 (6)
	South American	17 (8)
	Spanish or Spaniard	11 (5)
	Other	9 (4)
**Race**		
	White	249 (72)
	Asian	36 (10)
	More than one race	39 (11)
	Black or African American	18 (5)
	American Indian or Alaska Native	6 (2)
	Native Hawaiian or Pacific Islander	0 (0)
**Education**		
	Did not graduate from high school	7 (2)
	High school diploma or GED^a^	77 (22)
	Some college	104 (30)
	College degree	159 (46)

^a^GED: General Educational Development.

### Fitbit Data

**Figure 5:**
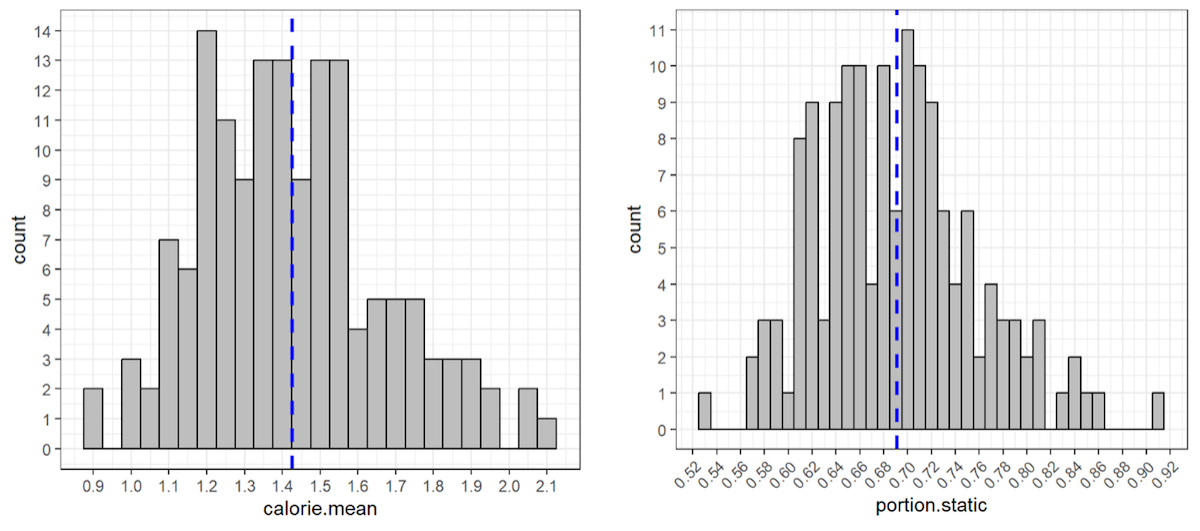
Histogram of mean calorie (left) and portion static (right).

To show between-subject variance in MetS-indicative daily behavior, we computed 2 example metrics—namely *mean calorie per minute* (calorie.mean) and *proportion of the time spent not moving* (portion.static)—for each of the 148 participants who returned at least 14 continuous days of data, over each person’s data collection period. We considered a given minute static if the difference between the current and the previous minute’s calorie expense was zero. Figure 5 shows the histogram of these two metrics; vertical, dashed blue lines indicate mean value. The average woman expended mean of 1.42 calories per minute (SD 0.24 calories), which translates to an average of 2048 calories per day (SD 348 calories), and remained static 69% of the time (SD 6.7%).

**Figure 6:**
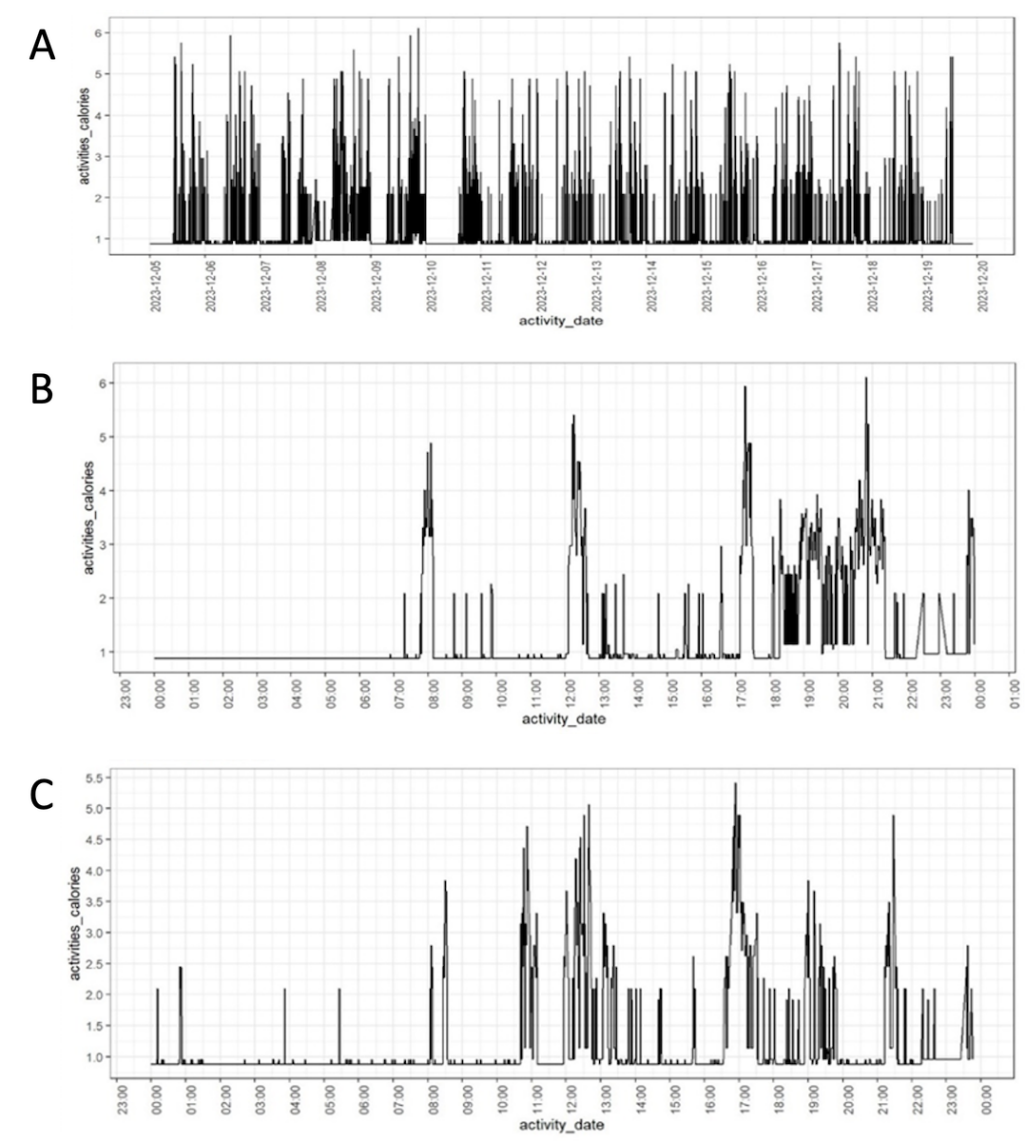
Time series of minute-by-minute energy expenditure of a participant: (A) over the entire data collection period, December 5–19, 2023; (B) December 9, 2023; and (C) December 13, 2023.

To illustrate within-person variance in energy expenditure, we plotted the time series of minute-by-minute calorie readings for a sample participant in Figure 6A, where the meaningful fluctuations reflect the daily cycle. Figures 6B and 6C are two different days extracted from the participant’s data collection period. Both exhibit an initial period of nonmovement, indicating sleep time, followed by activity spikes at various points throughout the day, which last for different durations.

### MRI Data

**Figure 7:**
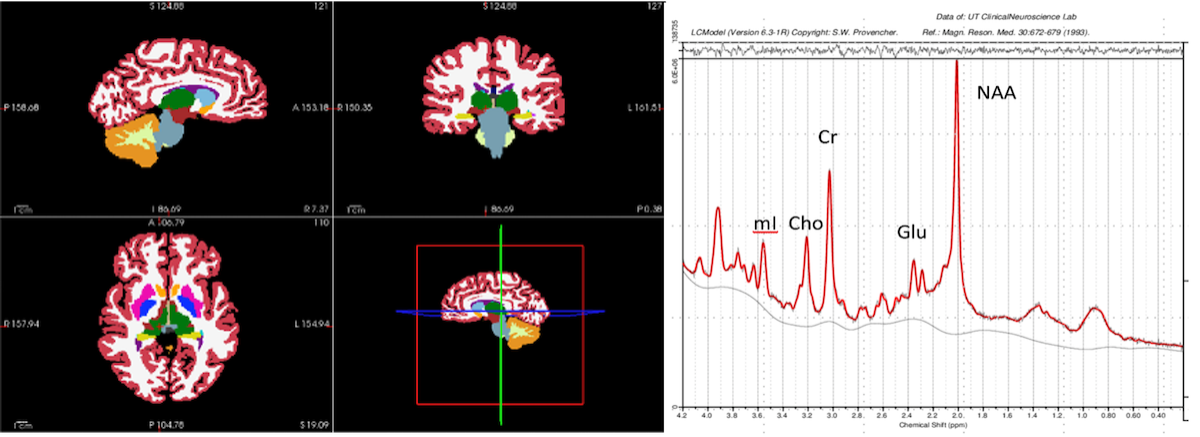
Magnetic resonance imaging brain measurements. FreeSurfer gray matter segmentation of T1-weighted magnetization-prepared rapid gradient-echo structural images (left). Linear Combination Model results from one participant’s magnetic resonance spectroscopy data (right). Cho: choline+phosphocholine; Cr: creatine+phosphocreatine; Glu: glutamate; mI: myo-inositol; NAA: N-acetylaspartate.

We have demonstrated the feasibility and tolerability of the MRI protocol by completing 47 scans. The representative results of the MRI segmentation and magnetic resonance spectrum are shown below, demonstrating excellent data quality (Figure 7).

### Air Quality Sensor Data

**Figure 8:**
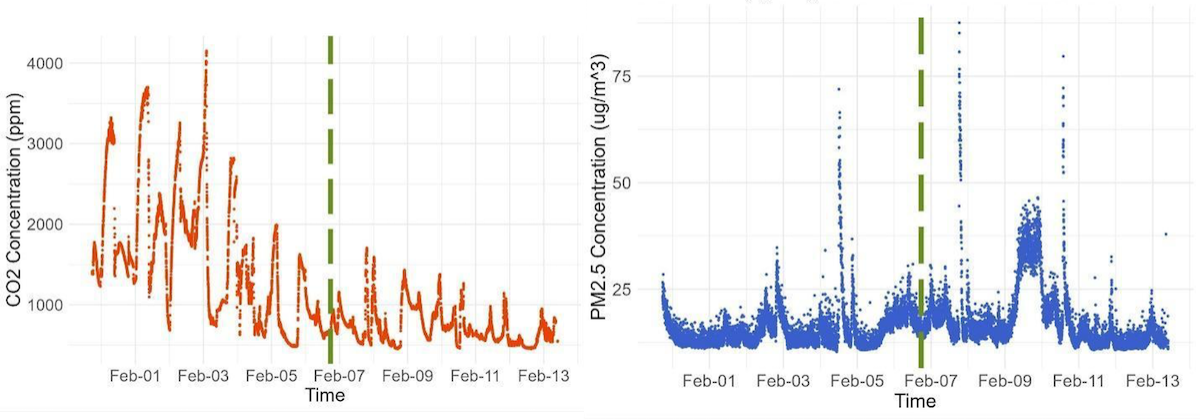
The home environment sensor kit measurements. Calibrated carbon dioxide (CO_2_; ppm) household indoor air measurements (left) and particulate matter ≤2.5 μm in diameter (PM_2.5_; μg/m³) household indoor air measurements. In the bedroom (week 1: left of the dashed axis), then the kitchen (week 2: right of the dashed axis).

We have successfully gathered CO_2_ and PM_2.5_ concentration measurements during the 2-week data collection period, as shown in Figure 8. PM_2.5_ levels increase during dinner time, hypothetically associated with the occurrence of cooking.

### Risk for Metabolic Syndrome

One aim of this study is to provide case examples of data from the first year of data collection, with the goal of promoting the standardization of measurements. This section presents 2 illustrative cases from the FEASible study, which evaluates risk for MetS.

#### Case 1: A High-Risk 39-Year-Old Non-Hispanic White Woman

At age 39, this participant is a single mother who struggles with sleep (less than 5 hours per night as measured by the Groningen Sleep Scale) because she cares for a teething toddler. She self-identifies as a non-Hispanic White woman with a background in performing arts, fine arts, and high school sports (eg, volleyball). She has a BMI of 44.8 kg/m^2^ and a waist circumference of 43 inches. Health screenings suggest she may have MetS and is at risk for CVD. Her common laboratory assessments reveal prehypertensive to hypertensive blood pressure (130/100 mm Hg), borderline high total cholesterol (206 mg/dL), and elevated triglycerides (231 mg/dL). Her self-reported health history revealed diagnosed fatty liver disease, frequent upper respiratory infections, and irregular thyroid function. She lives in a neighborhood with no sidewalks and limited access to healthy food options (Neighborhood Factors Questionnaire). Further, this participant reported experiencing frequent discrimination related to her weight (245 lb) and gender (Everyday Discrimination Scale). Despite working as a nanny, which involves some days of moderate physical activity (self-reported International Physical Activity Questionnaire), she confirmed that she does not meet the weekly or recommended amount of physical activity duration, steps, or intensity. Although she is currently receiving health care, she is at significant risk of a prolonged poor health span. This participant represents our standard practice for addressing health risks: wait for symptomatology, then seek urgent or emergency care. In contrast, our second case reflects the potential for early intervention among young adults. By using wearable devices and home sensors, we can develop statistical models that link the magnitude of risk and identify points of intervention (eg, home exposures and physical activity).

#### Case 2: 20-Year-Old Hispanic Woman

The second case involves a 20-year-old Hispanic woman, born in Mexico, who resides in Texas in the same neighborhood as the first case. While she faces similar environmental challenges, including outdoor noise and a lack of access to sidewalks, parks, and healthy foods (as indicated by the Home Air Quality and Neighborhood Factors Questionnaire), her health profile differs significantly. She reports sleeping well, averaging 8.5 hours of sleep per night (as measured by the Groningen Sleep Scale, Ecological Momentary Assessments, and Fitbit data). Her BMI of 26.8 kg/m^2^ places her in the overweight category, but all other biomarkers are within healthy ranges. According to the International Physical Activity Questionnaire, she spends most of her awake time sitting, with occasional walking but limited moderate-to-vigorous physical activity. Fitbit data provide further evidence of low activity levels. Over the 17-day observation period (July 8-24, 2024), her average daily energy expenditure was 1882 calories (1.31 calories per minute), compared to the cohort average 2048 calories, indicating lower-than-average activity levels. However, her average daily static percentage was 68% (percentage of time spent not moving during a 24-hour period), no higher than the cohort average of 67%. 

By comparing these two cases, which represent mid-life and emerging adult developmental stages, we aim to refine algorithms for estimating MetS risk and brain vulnerability. This approach will help identify behavioral and environmental variables contributing to health disparities, enabling the development of tailored and responsive interventions.

## Discussion

### Overview

The FEASible study explores women’s daily living patterns through wearable devices and home environment sensor kit to validate the use of behavioral and environmental data (eg, physical activity, sleep, and indoor air quality) as indicators of risk for MetS and CVD (National Heart, Lung, and Blood Institute; grant R01HL168374). This investigation is designed to provide a rationale for the standard operating procedures and provide a case example of data from the first year of data collection leading to artificial intelligence and machine learning modeling of health risk, thus informing future interventions focused on MetS, heart disease, and all-cause mortality among Latina women. This study consists of three steps on data collection from participants for one month: (1) self-reported demographics, health, and lifestyle; (2) health screening and toolkit deployment with sensors for 2-week data sensing; and (3) neuroimaging and blood draw. The preliminary results of this study provide valuable insights into the participants’ demographics and the progress of data collection. Findings are at an initial stage. Nevertheless, the study has successfully recruited a diverse pool of participants, with a particular focus on Latina women. The primary conclusion of this study is that, upon completion of the first year of data collection, FEASible is feasible, has been implemented as intended, and is ready to scale up***.***

The participants recruited are diverse in terms of age groups, ethnicities, races, and educational backgrounds. Most participants fall within the age range of 18-25 years, which aligns with the study’s focus on young adults. However, the lower representation of participants aged 34-40 years warrants targeted recruitment efforts to ensure adequate representation of this age group in the final sample. The most helpful recruitment strategy has been word of mouth among participants. Participants share the study with friends and relatives within their communities, including the University of Texas at Austin. However, consistent with previous studies, recruitment by word of mouth rarely achieves a high sample size, highlighting the need for alternative recruitment strategies [37]. Another recruitment strategy has been posting about the study in Latina-focused Facebook groups, which has helped attract more participants from diverse educational and age backgrounds. Posting on existing Facebook groups and pages has efficiently generated responses from potential participants, particularly since the COVID-19 pandemic [38]. Another recruitment strategy will be to work directly with a community-based health clinic and volunteer at community events.

Community engagement is a valuable asset for this study, as our ultimate goal is to develop potential interventions to address future challenges related to CVD and MetS. Community engagement must be a two-way exchange process between the university and its communities, aiming to facilitate transparent and ongoing communication for study participants [39]. Therefore, the research team will continue to volunteer within the community, offering potential participants talks on CVD and brain health, as well as resources. Our dissemination plan involves providing health screening assessment letters to participants, which they can share with their primary health care practitioner. Moreover, participants can track their physical activity and sleep patterns using wearable devices and obtain preliminary information about the air quality in their homes.

The proportional representation of Latina and non-Latina participants has numerous advantages, as it aims to address the unique challenges and health concerns this population faces. Moreover, the diversity of Latin origin, with participants from various regions, further enriches the findings and provides an opportunity to evaluate the Latina population as disaggregated data, illuminating the community’s diversity. This level of granularity is crucial, as previous research has highlighted significant heterogeneity in health outcomes and CVDs and MetS risk factors among different Hispanic and Latino subgroups [40-42]. By capturing this diversity, the study can contribute to a more nuanced understanding of the complex interplay between cultural, environmental, and behavioral factors influencing cardiovascular and metabolic health in Latina women.

Sensing devices address a critical need for cost-effective, accessible, objective, and continuous monitoring of health-related factors. Poor physical activity and sleep patterns have been consistently associated with an increased risk of MetS in women [43]. However, the association of environmental factors, such as air quality and indoor pollutants, on cardiovascular and cognitive health has been understudied, particularly in Latina women.

It is important to note that the FEASible study is cross-sectional in design, which cannot establish causal relationships between behavioral or environmental factors and MetS. Findings can only identify associations between these variables. Future longitudinal and experimental studies are planned to examine temporal and causal pathways linking lifestyle behaviors, environmental exposures, and cardiometabolic outcomes.

The study’s findings have the potential to inform future experimental or clinical studies investigating causal relationships and developing targeted interventions for CVDs and MetS prevention among Latina women. Additionally, validating low-cost and accessible monitoring devices may pave the way for their integration into clinical practices, enabling personalized and continuous monitoring of health risks.

### Conclusions

The FEASible study addresses a critical gap in understanding the complex interplay of factors contributing to CVDs and MetS among Latina women, a population disproportionately affected by these conditions. The comprehensive approach to evaluating physical activity, sleep patterns, and environmental factors aligns with the need for tailored prevention strategies. Using low-cost sensing devices is a promising approach to capturing real-world data, but validation is necessary to assess reliability and accuracy to ensure the quality and interpretability of the collected data. Moreover, this study emphasizes the importance of developing affordable and precise devices to accurately monitor individual behaviors and environmental risk factors, thereby enabling modeling approaches that assess health risks. It can aid the development of “sequential multiple assignment randomized trials” interventions to address MetS and CVDs, as well as their cognitive consequences.

## Appendix

Multimedia Appendix 1 Peer review report from the CIDH - Clinical Informatics and Digital Health Study Section, Healthcare Delivery and Methodologies Integrated Review Group (National Institutes of Health, USA).

## Abbreviations

BOLD fMRI: blood oxygenation level–dependent functional MRI
CO_2_: carbon dioxide
CVD: cardiovascular disease
FLAIR: fluid-attenuated inversion recovery
FOV: field of view
MetS: metabolic syndrome
MPRAGE: magnetization prepared rapid gradient echo
MRI: magnetic resonance imaging
MRS: magnetic resonance spectroscopy
pcASL: pseudo-continuous arterial spin labeling
PM_2.5_: particulate matter ≤2.5 μm in diameter
REDCap: Research Electronic Data Capture
RRAMSC: relaxation-resolved advanced multi–spin-echo chemical shift
SE: spin echo
SVS: single voxel spectroscopy
TA: total acquisition time
TE: echo time
TI: inversion time
TR: repetition time

## Data Availability

The data collection of the FEASible Study is still in progress. The datasets generated during this study are not publicly available. Interested researchers may contact the Principal Investigators to request access. Data requests will be reviewed on a case-by-case basis following NIH guidelines and participant confidentiality protections.
