# Supplementary material for: Assessing Associations Between Environmental, Sleep, and Physical Activity Factors and Metabolic Syndrome Risk: Protocol for the FEASible Study
Source: JMIR Res Protoc. 2026 Jan 6;15:e82034. doi: 10.2196/82034 (PMC12820547; doi:10.2196/82034)
Supplement: Multimedia Appendix 1 [file resprot_v15i1e82034_app1.pdf]

SUMMARY STATEMENT
(Privileged Communication)

PROGRAM CONTACT:
ALISON Brown
301-435-0583
alison.brown@nih.gov

Release Date: **10/21/2022**
Revised Date:

Application Number: **1 R01 HL168374-01**

Principal Investigators (Listed Alphabetically):

CASTELLI, DARLA M (Contact)
HALEY, ANDREANA PETROVA

Applicant Organization: UNIVERSITY OF TEXAS AT AUSTIN

Review Group: **CIDH**
Clinical Informatics and Digital Health Study Section

Meeting Date: **09/29/2022**
Council: **JAN 2023**
Requested Start: **01/01/2023**
RFA/PA: **PA20-185**
PCC: **SHCBMN**

Project Title: **FEASible: Sensing Factors of Environment, Activity, and Sleep to Validate Metabolic Health Burden Among Latina Women**
SRG Action: **Impact Score:31 Percentile:13**
Next Steps: **Visit https://grants.nih.gov/grants/next_steps.htm**
Human Subjects: 48-At time of award, restrictions will apply
Animal Subjects: 10-No live vertebrate animals involved for competing appl.
Gender: 2A-Only women, scientifically acceptable
Minority: 1A-Minorities and non-minorities, scientifically acceptable
Age: 7A-Only Adults, scientifically acceptable

Project Year	Direct Costs Requested	Estimated Total Cost
1	499,930	764,422
2	499,988	764,511
3	499,982	764,502
4	499,284	763,434
5	431,596	659,935
TOTAL	2,430,780	3,716,804

ADMINISTRATIVE BUDGET NOTE: The budget shown is the requested budget and has not been adjusted to reflect any recommendations made by reviewers. If an award is planned, the costs will be calculated by Institute grants management staff based on the recommendations outlined below in the COMMITTEE BUDGET RECOMMENDATIONS section.

1R01HL168374-01 Castelli, Darla

PROTECTION OF HUMAN SUBJECTS UNACCEPTABLE

RESUME AND SUMMARY OF DISCUSSION: This application proposes to measure metabolic syndrome (Met-S) and Met-S related brain vulnerability of Latino Women and associate these with environmental exposures (indoor/outdoor) and mobile sensor data of daily behavior (activity, heart rate, sleep) to develop validated proxy measures of Met-S. The review panel agreed with the investigators regarding the significance of the application's focus on metabolic syndrome among Latino populations given the high prevalence of Met-S and potential for cognitive impairment, morbidity, and mortality. The ability to measure risk factors by using cheaper and more widely deployed sensors was viewed as contributing to the significance of the application. The PI was noted has having significant experience in related research, supported by a broad range of experts in multiple areas required for successful completion of the aims (clinical psychology, imaging, engineering, health disparities, etc.). The addition of a biostatistician was thought as needed to strengthen the application's analysis and rigor. The application's multisensory-based data collection, including air quality monitoring, was noted as an important innovation, as well as a strength of the research strategy. The panel did highlight some notable weaknesses in the approach, including an ambitious sample for home-based sensor and air quality data collection, the environmental monitoring that was limited to at-home (where participations may or may not spend a significant amount of time), as well as insufficient information about the machine learning models to be leveraged for understanding Met-S risks. The panel discussion centered on whether the outcome of Met-S related brain vulnerability was valid and useful. While some on the panel noted that the MRI derived data were acceptable, although not perfect outcome measures, others on the panel assessed that MRI derived data was being treated as a gold standard that was not widely clinically accepted. They raised alternatives, such as CMR02, that were thought to be more appropriate for assessing brain vulnerability. Overall, while some on the panel weighed the weaknesses to a greater degree, the predominant majority assessed that due to strengths in the significance, investigators, and innovation the application would have a high impact for furthering the understanding at Met-S, with insights to the multiplicity of factors that impacted metabolic health among Latino women.

DESCRIPTION (provided by applicant): Passive sensing of Latina women's daily living experience as proxy measures of health risk must be brought to scale. We propose to validate the integrated measurements from Fitbits, smartphones, purpose-built environmental sensors (Beacon), GPS, and a purpose-built smartphone app (Hornsense), to assess physical activity, sleep, and environmental exposures as they relate to metabolic syndrome (MetS) and MetS-related brain vulnerability. We will develop a pipeline to create and interpret networks signifying how MetS relate to brain integrity in Latina women and identify which environmental factors contribute to increased risk for MetS. To achieve these goals, we synergize expertise from multiple disciplines, advance pilot data, and ground-truth novel integrated measures. First, it is essential to define how MetS risk manifests in this sample by identifying how MetS affects brain integrity in a subsample of Latina women compared to white women (n=225) and compare these methods to traditional clinical assessments of MetS (defined by AHA/NHLBI). Measures of cerebral metabolism (N-acetyl aspartate, NAA, myo-inositol, mI and glutamate, Glu) will form a network of risk and brain vulnerability. Daily living will be assessed in 1,000 women (60% Latina) through 30 days of dense sensing in the home environment. Participants will wear Fitbit Inspire devices and have the Hornsense app on their cell phones. Environmentally, we will validate the purpose-built Beacon, which measures environmental factors of particulate matter (PM2.5,- allergens that influence air quality), nitrogen oxides (NOx - gases within smog), carbon monoxide (CO) and carbon dioxide (CO2), temperature, relative humidity (RH), and amount of noise, first against research-grade reference instruments in well-controlled sleep chambers and smart test homes, and second in participant homes. During the dense sensing, Beacons and Personal Air Quality Monitor (PAM) will be placed in the bedroom and outside the home for one month. The 24 hours of activity, location, and all environmental values will be compared with MetS risk and MetS-related brain vulnerability because brain integrity is

among the earliest markers of vulnerability, representing a high risk for poor health outcomes and quality of life in older age. Network analysis of these data will identify the critical central nodes of risk. Scaling our pilot sensing protocols will determine the feasibility and efficacy of integrated measurements of daily behavior, activity, sleep, and environmental attributes to predict MetS and MetS-related brain vulnerability in Latina women. Identifying valid strategies for targeting individual behaviors and contexts is essential for future prevention efforts to be designed with greater precision, particularly for Latina women.

PUBLIC HEALTH RELEVANCE: This project will validate low-cost mobile sensors (smartphones, smartwatches, home-based environmental sen- sors) to capture sleep, physical activity, location, and environmental hazards in and around the homes of 1000 Latina women as proxy measures of metabolic syndrome (MetS) risk. The sensing data will be ground-truthed using clinical measures of MetS and models of MetS-related brain vulnerability networks among a subset of participants (n=225). Our transdisciplinary approach uses gold-standard clinical measures to validate integrated measuremnets of mobile sensing devices as a cluster proxy assessment of MetS risk and neuroimaged MetS- related brain vulnerability.

CRITIQUE 1

Significance: 2
Investigator(s): 2
Innovation: 3
Approach: 3
Environment: 2

Overall Impact: In the US, the prevalence of metabolic syndrome (MetS) has increased 35% over the past two decades and Latina women have the highest prevalence in the US. However, little is known about the high prevalence of MetS in Latino women other than information from self-reported surveys. MetS has been linked to cognitive impairment, cardiovascular disease, and all-cause mortality. Hence, better understanding of the causes of MetS, especially in underrepresented minorities such as Latina women, is highly needed. To this end, this work proposes to determine which modifiable peripheral cardiometabolic biomarkers are associated with MetS-related brain vulnerability and use them as the ground-truth standards in their Aim 1. The second aim is to measure environmental exposures that can detrimentally affect the quality of sleep and physical activity by measuring air pollutants in the participants' homes using a low-cost Beacon device the investigative team has previously developed and tested. The final aim is to determine the feasibility and efficacy of mobile sensor measurements of daily behavior, activity, sleep, and environmental attributes to predict MetS and MetS-related brain vulnerability. This is a well written proposal with the proposed aims well supported by the investigative team's prior research findings and the previous development and testing of Beacon device which will be used to measure air quality in participants' homes. The main goal is to better understand MetS and MetS-related brain vulnerability in Latina women as they have the most prevalence and yet are the least studied population. In addition, the main strength of this proposal is the multi-faceted sensor-based data collection approach which will provide a more accurate determinant of MetS and MetS-related brain vulnerability when compared to the traditional approach of using only actigraphy measurements. Given that this proposal is well grounded by the investigators' prior experience in this area of work, demonstrated by ample preliminary data, and that the study will rely on multi-faceted sensor measurements, the likelihood of success and the impact of this work will be quite high.

1. Significance:
Strengths

- Prior development of a low-cost Bevo Beacon device for measuring several key metrics of air quality is one of the key strengths.
- Examining MetS and MetS-related brain vulnerability in Latinas as they have the most prevalence and yet are the least studied population
- Multi-faceted sensor data collection consisting of MRI, smart sensor data, and air quality measurements will lead to better quantification and prediction of MetS and MetS-related brain vulnerability in Latina women.

Weaknesses

- This study is ambitious in that it will examine 1,000 Latina women of which neuroimaging will be done in a subsample of 225 participants. Getting wearable sensor and air quality data from the Beacon device and other gold-standard devices for comparison to the former from 1,000 participants may be challenging. However, if data collection is obtained from these 1,000 participants, the likelihood of success is even greater.

2. Investigator(s):

Strengths

- The PI, Dr. Castelli, has over 30 years of experience in human development, cognition, and health disparity research and is an ideal leader for the proposed work. The team's other researchers include expertise in clinical psychology including neuroimaging (Dr. Haley), systems engineering (Dr. Wu), metabolic syndrome research (Dr. Schnyer), chemical engineering and environmental engineering with expertise in air quality research (Drs. Kinney and Maestre), and exercise physiology (Dr. Harrison). There is also an outreach coordinator (Ms. Garcia). This investigative team is complete with proper expertise and some of the investigators have worked and published together in the past.

Weaknesses

- A biostatistician is missing but would be useful given the significant amount of data that will be collected from 1,000 subjects.

3. Innovation:

Strengths

- Inclusion of direct environmental and daily activity sensing in MetS research, as previous studies did not consider spatial, temporal, and cultural relevance
- Concurrent examination of data related to MetS criteria and brain vulnerability and daily living monitoring
- Prior development of Bevo Beacon which is more cost-effective than commercially available devices, which will be deployed for measuring air quality in participants' homes. The device has been tested in prior work so only calibration to the commercially-available device needs to be done.

Weaknesses

- Machine learning, network analysis, and analysis to identify combinations of features related to health risk will involve the standard programs.

4. Approach:

Strengths

- The inclusion age limit >= 29 years and women of child-bearing age and exclusion of midlife individuals to focus on behaviors that can be modified before post-menopausal effects and age-related cognitive decline is well planned to mitigate other confounding factors.
- The use of a low-cost Beacon device and testing it against commercially-available air quality determinant devices is sound and improves the feasibility of deploying it in nearly 1,000 subjects' homes.
- The use of multiple sensors including air quality and activity and social network data combined with MRI data will provide more in-depth and comprehensive understanding of factors and features that may link to MetS and Mets-related brain vulnerability.
- If the study is successful, and it seems to have all the necessary ingredients for positive outcomes, it will lead to an intervention trial to improve brain vulnerability in Latina women for the follow-up study. Hence, there is direction to the future path for this work.

Weaknesses

- It is not clear if 30-day sensing of behavior and environmental exposures is sufficient to elicit changes in MetS and MetS-related brain vulnerability.
- Collection of MRI data and subsequent data analysis from 225 participants might be overly optimistic given the logistical and cost challenges.
- Details regarding machine learning, feature extraction, and network analysis are rather succinct but since these are based on the standard approaches, this is a minor concern.

5. Environment:

Strengths

- The PI and the investigative team have established labs and are associated with facilities to perform the proposed work. There is a dedicated Biomedical Imaging Center at UT Austin for the proposed MRI data collection. Latina subjects will be recruited through the Whole Communities – Whole Health center located at UT Austin.

Weaknesses

- None

Study Timeline:

Strengths

- None noted by Reviewer

Weaknesses

- None noted by Reviewer

Protections for Human Subjects:

Acceptable Risks and/or Adequate Protections

- Other than blood draw, all data collection involves non-invasive measurements. All subjects will be presented with an informed consent form with description of the study's potential risks and benefits.

Data and Safety Monitoring Plan (Applicable for Clinical Trials Only):

Not Applicable (No Clinical Trials)

Inclusion Plans:

- Sex/Gender: Distribution justified scientifically
- Race/Ethnicity: Distribution justified scientifically
- For NIH-Defined Phase III trials, Plans for valid design and analysis:
- Inclusion/Exclusion Based on Age: Distribution justified scientifically
    - The study will focus on Latina women as they have the most prevalence of MetS and they have been traditional the least-studied. Inclusion and exclusion criteria based on age are well justified.

Vertebrate Animals:

Not Applicable (No Vertebrate Animals)

Biohazards:

Not Applicable (No Biohazards)

Resource Sharing Plans:

Not Applicable (No Relevant Resources)

Authentication of Key Biological and/or Chemical Resources:

Not Applicable (No Relevant Resources)

Budget and Period of Support:

Recommend as Requested

CRITIQUE 2

Significance: 1
Investigator(s): 1
Innovation: 2
Approach: 3
Environment: 1

Overall Impact:

1. **Significance:**

Strengths

- MetS-related disease is in importance healthcare issue, particularly in the Latina population, which is targeted in this grant
- This proposal would collect dense IAQ, activity and brain quality metrics that would likely be valuable in understanding MetS

Weaknesses

- None noted by Reviewer

2. Investigator(s):

Strengths

- Much experience in population engagement and measurement of physiologic variables associated with health, particularly in Hispanic population.
- They have also previously collected activity, IAQ, and MRI measurements for other research

Weaknesses

- None noted by Reviewer

3. Innovation:

Strengths

- Strong focus on a specific population and calibration of cheaper devices

Weaknesses

- What they propose has largely been done before, just not with this population focus, and with more pervasive environment sensing.

4. Approach:

Strengths

- The investigators have done nearly all of the pieces proposed in the past, just not at the density nor with the population target proposed. As such, this is highly likely to achieve its goals.
- The methods are well described, likely because they have done all the pieces before.

Weaknesses

- The investigators do not describe how they will address privacy of the data, particularly since (to their credit) the data will be made available. Will GPS coordinates be encoded in some way?
- The sampling strategy for environmental factors may be too sparse to be viable.
- There is no comment on the ability to recruit subjects. They have done large studies before, but this is a unique population that may be more wary than college students. Some comment on this would seem warranted.
- What does it mean that 'all data will be imputed' page 105 (C.6)? Imputation is for filling in missing values. If that is the correct meaning, t(which is reasonable—sensors may miss sending a given data point) he method of imputation should be described.

5. Environment:

Strengths

- The investigators have done all the pieces before, so execution risk is low.

Weaknesses

- None noted by Reviewer

Study Timeline:

Strengths

- None noted by Reviewer

Weaknesses

- None noted by Reviewer

Protections for Human Subjects:

Unacceptable Risks and/or Inadequate Protections

- More description of data privacy is needed

Inclusion Plans:

- Sex/Gender: Distribution justified scientifically
- Race/Ethnicity: Distribution justified scientifically
- For NIH-Defined Phase III trials, Plans for valid design and analysis: Scientifically acceptable
- Inclusion/Exclusion Based on Age: Distribution justified scientifically
    - They focus on female Hispanics because that population is currently understudied, so the focus on that group is acceptable. What is not clearly acceptable is the lack of description of how identifiable data about subjects will be handled, and steps that will be taken to mitigate privacy concerns.

Vertebrate Animals:

Not Applicable (No Vertebrate Animals)

Biohazards:

Not Applicable (No Biohazards)

Resource Sharing Plans:

Acceptable

- Description of how de-identification will be accomplished is needed.

Authentication of Key Biological and/or Chemical Resources:

Not Applicable (No Relevant Resources)

Budget and Period of Support:

Recommend as Requested

CRITIQUE 3

Significance: 2
Investigator(s): 2
Innovation: 4
Approach: 4
Environment: 1

Overall Impact: This is a new R01 submission by Drs. Darla Castelli and Andreana Haley of UT Austin and colleagues from the same institution and Binghamton University. The project is to develop a framework to understand metabolic syndrome (MetS) relate to brain integrity in Latina women and identify which environmental factors contribute to increased risk for MetS using various sensors and 3T MRI images. MetS has increased 35% over the past two decades and among the population suffering from MetS, Latina women have the highest prevalence. Research to provide better care to this underserved population is significant. The research team has existing collaborations and is well-qualified to conduct the research. The concerns include (1) environmental quality measurements are all for in-home settings. This assumption excludes those subjects who spend a significant amount of time in one or more working locations. (2) Considering the complexity of sensor signals, MRT images, and other clinical attributes, more concrete methods in machine learning are needed for in-depth understanding of the MetS risks for certain subgroups of subjects under specific environments. (3) There are notable dependencies between aims.

1. Significance:

Strengths

- MetS has increased 35% over the past two decades and among the population suffering from MetS, Latina women have the highest prevalence. Research to provide insights to understand potential causes from their daily living for this underserved population is significant.

Weaknesses

- None

2. Investigator(s):

Strengths

- Drs. Darla Castelli and Andreana Haley of UT Austin and other senior personnel are well qualified to conduct the MetS research.

Weaknesses

- Machine learning expertise is absent from the research team. This reflects on the weakness in data analytics and translating data-driven results to the “bedside”.

3. Innovation:

Strengths

- Measuring a broad range of environmental factors of daily living using various sensors to study MetS is novel.

Weaknesses

- The linkages between the ground truth from MRI markers to the large variety of environmental factors are not emphasized. This weakens the clinical relevance of the project outcomes.

4. Approach:

Strengths

- The proposal has a reasonable plan from subject recruitment, sensor study, marker assessments.

Weaknesses

- Environmental quality measurements are all for in-home settings. This assumption excludes those subjects who spend a significant amount of time in one or more working locations.
- Considering the complexity of sensor signals, MRT images, and other clinical attributes, more concrete methods in machine learning are needed for in-depth understanding of the MetS risks for certain subgroups of subjects under specific environments.
- There are notable dependencies between Aim 1 & Aim 3 and Aim 2 and Aim 3.

5. Environment:

Strengths

- UT Austin has an excellent research environment for the proposed project.

Weaknesses

- None

Study Timeline:

Strengths

- N/A

Weaknesses

- N/A

Protections for Human Subjects:

Acceptable Risks and/or Adequate Protections

- The project has a comprehensive plan for human subject protection.

Data and Safety Monitoring Plan (Applicable for Clinical Trials Only):

Not Applicable (No Clinical Trials)

Inclusion Plans:

- Sex/Gender: Distribution justified scientifically
- Race/Ethnicity: Distribution justified scientifically
- For NIH-Defined Phase III trials, Plans for valid design and analysis:
- Inclusion/Exclusion Based on Age: Distribution justified scientifically
  - The project focuses on Latina women with ages 29-40 which is justified.

Vertebrate Animals:

Not Applicable (No Vertebrate Animals)

Biohazards:
Not Applicable (No Biohazards)

Resource Sharing Plans:
Acceptable

Authentication of Key Biological and/or Chemical Resources:
Not Applicable (No Relevant Resources)

Budget and Period of Support:
Recommend as Requested

THE FOLLOWING SECTIONS WERE PREPARED BY THE SCIENTIFIC REVIEW OFFICER TO SUMMARIZE THE OUTCOME OF DISCUSSIONS OF THE REVIEW COMMITTEE, OR REVIEWERS' WRITTEN CRITIQUES, ON THE FOLLOWING ISSUES:

PROTECTION OF HUMAN SUBJECTS: UNACCEPTABLE: The panel found the human subjects considerations did not sufficiently handle the privacy risks to subjects.

INCLUSION OF WOMEN PLAN: ACCEPTABLE

INCLUSION OF MINORITIES PLAN: ACCEPTABLE

INCLUSION ACROSS THE LIFESPAN: ACCEPTABLE

COMMITTEE BUDGET RECOMMENDATIONS: The budget was recommended as requested.

Footnotes for 1 R01 HL168374-01; PI Name: Castelli, Darla M

NIH has modified its policy regarding the receipt of resubmissions (amended applications).See Guide Notice NOT-OD-18-197 at https://grants.nih.gov/grants/guide/notice-files/NOT-OD-18-197.html. The impact/priority score is calculated after discussion of an application by averaging the overall scores (1-9) given by all voting reviewers on the committee and multiplying by 10. The criterion scores are submitted prior to the meeting by the individual reviewers assigned to an application, and are not discussed specifically at the review meeting or calculated into the overall impact score. Some applications also receive a percentile ranking. For details on the review process, see http://grants.nih.gov/grants/peer_review_process.htm#scoring.

MEETING ROSTER

Clinical Informatics and Digital Health Study Section
Healthcare Delivery and Methodologies Integrated Review Group
CENTER FOR SCIENTIFIC REVIEW
CIDH
09/29/2022 - 09/30/2022

Notice of NIH Policy to All Applicants: Meeting rosters are provided for information purposes only. Applicant investigators and institutional officials must not communicate directly with study section members about an application before or after the review. Failure to observe this policy will create a serious breach of integrity in the peer review process, and may lead to actions outlined in NOT-OD-22-044 at https://grants.nih.gov/grants/guide/notice-files/NOT-OD-22-044.html, including removal of the application from immediate review.

CHAIRPERSON(S)

DEXHEIMER, JUDITH W, PHD
ASSOCIATE PROFESSOR
DEPARTMENT OF PEDIATRICS
AND BIOMEDICAL INFORMATICS
CINCINNATI CHILDREN'S HOSPITAL MEDICAL CENTER
CINCINNATI, OH 45229

MEMBERS

ABDULLAH, SAEED, PHD *
ASSISTANT PROFESSOR
COLLEGE OF INFORMATION SCIENCES AND TECHNOLOGY
PENNSYLVANIA STATE UNIVERSITY
UNIVERSITY PARK, PA 16802

ALPERN, ELIZABETH RACHEL, MD
PROFESSOR
DEPARTMENT OF PEDIATRICS
ANN AND ROBERT H. LURIE CHILDREN'S HOSPITAL
FEINBERG SCHOOL OF MEDICINE
NORTHWESTERN UNIVERSITY
CHICAGO, IL 60611

BARTLETT, CHRISTOPHER WILIAM, PHD *
ASSOCIATE PROFESSOR OF PEDIATRICS
DEPARTMENT OF PEDIATRICS/ BATTLE CENTER FOR
MATHEMATICAL MEDICINE
NATIONWIDE CHILDREN'S HOSPITAL
COLUMBUS, OH 43205

CHON, KI H, PHD
PROFESSOR
DEPARTMENT OF BIOMEDICAL ENGINEERING
UNIVERSITY OF CONNECTICUT
STORRS, CT 06269

CHUNG, JANE, PHD *
ASSISTANT PROFESSOR
SCHOOL OF NURSING
VIRGINIA COMMONWEALTH UNIVERSITY
RICHMOND, VA 23298

DIAS, ROGER DAGLIUS, PHD, MD, MBA *
ASSISTANT PROFESSOR, DIRECTOR OF RESEARCH AND
INNOVATION, STRATUS CENTER FOR MEDICAL
SIMULATION, DIRECTOR AND LEAD INVESTIGATOR, HUMAN
FACTORS AND COGNITIVE ENGINEERING LAB
DEPARTMENT OF EMERGENCY MEDICINE
HARVARD UNIVERSITY
BOSTON, MA 02115

ERICKSON, BRADLEY J, PHD *
PROFESSOR
DEPARTMENT OF RADIOLOGY
MAYO CLINIC
ROCHESTER, MN 55905

FENTON, SUSAN HRACHOVY, PHD, MBA *
ASSOCIATE PROFESSOR
SCHOOL OF BIOMEDICAL INFORMATICS
UNIVERSITY OF TEXAS HEALTH SCIENCE CENTER
HOUSTON, TX 77030

FRONTERA, JENNIFER ANN, MD *
PROFESSOR
DEPARTMENT OF NEUROLOGY
NEW YORK GROSSMAN SCHOOL OF MEDICINE
NEW YORK, NY 10016

GRUNDMEIER, ROBERT W, MD
DIRECTOR OF CLINICAL INFORMATICS
DEPARTMENT OF BIOMEDICAL AND HEALTH INFORMATICS
CHILDREN'S HOSPITAL OF PHILADELPHIA
PHILADELPHIA, PA 19146

GUO, JINGCHUAN, PHD *
ASSISTANT PROFESSOR
DEPARTMENT OF PHARMACEUTICAL OUTCOMES AND
POLICY
COLLEGE OF PHARMACY
UNIVERSITY OF FLORIDA
GAINESVILLE, FL 32610

HATEF-NAIMI, ELHAM, MD, MPH *
ASSISTANT PROFESSOR
CENTER FOR POPULATION HEALTH IT
DEPARTMENT OF HEALTH POLICY AND MANAGEMENT
SCHOOL OF PUBLIC HEALTH
JOHNS HOPKINS UNIVERSITY
BALTIMORE, MD 21205

JEFFERY, ALVIN DEAN, PHD, RN *
ASSISTANT PROFESSOR
DEPARTMENT OF BIOMEDICAL INFORMATICS
SCHOOL OF NURSING
VANDERBILT UNIVERSITY
NASHVILLE, TN 37240

JORDAN, JENNIFER HAWTHORNE, PHD *
ASSISTANT PROFESSOR
DEPARTMENT OF BIOMEDICAL ENGINEERING
AND PAULEY HEART CENTER
VIRGINIA COMMONWEALTH UNIVERSITY
RICHMOND, VA 23284

KAMALESWARAN, RISHIKESAN, PHD *
DIRECTOR OF TRANSLATIONAL CLINICAL INFORMATICS,
ASSISTANT PROFESSOR
DEPARTMENT OF BIOMEDICAL INFORMATICS
PEDIATRICS, AND EMERGENCY MEDICINE
EMORY UNIVERSITY SCHOOL OF MEDICINE
ATLANTA, GA 30322

KENT, DAVID M, MD, CM *
PROFESSOR OF MEDICINE, NEUROLOGY AND CLINICAL
AND TRANSLATIONAL SCIENCE, DIRECTOR, PREDICTIVE
ANALYTICS AND COMPARATIVE EFFECTIVENESS (PACE)
CENTER
PREDICTIVE ANALYTICS AND COMPARATIVE
EFFECTIVENESS CENTER
INSTITUTE FOR CLINICAL RESEARCH HEALTH POLICY
TUFTS MEDICAL CENTER
BOSTON, MA 02111

KOVELL, LARA, MD *
ASSISTANT PROFESSOR
DEPARTMENT OF MEDICINE
CHAN MEDICAL SCHOOL
UNIVERSITY OF MASSACHUSETTS
WORCESTER, MA 01655

LIU, FEIFAN, PHD *
ASSISTANT PROFESSOR
DEPARTMENT OF POPULATION
AND QUANTITATIVE HEALTH SCIENCES
CHAN MEDICAL SCHOOL
UNIVERSITY OF MASSACHUSETTS
WORCESTER, MA 01605

LYLES, COURTNEY REES, PHD
ASSOCIATE PROFESSOR
DEPARTMENT OF MEDICINE
UNIVERSITY OF CALIFORNIA, SAN FRANCISCO
SAN FRANCISCO, CA 94110

MAHAJAN, AMAN, PHD *
PETER AND EVA SAFAR PROFESSOR AND CHAIR,
PROFESSOR OF BIOENGINEERING AND BIOMEDICAL
INFORMATICS
DEPARTMENT OF ANESTHESIOLOGY AND
PERIOPERATIVE MEDICINE
UNIVERSITY OF PITTSBURGH
PITTSBURGH, PA 15261

MARQUINE, MARIA, PHD *
ASSOCIATE PROFESSOR
DEPARTMENT OF MEDICINE, GERIATRICS DIVISION
DUKE CENTER FOR THE STUDY OF AGING
AND HUMAN DEVELOPMENT
DUKE UNIVERSITY SCHOOL OF MEDICINE
DURHAM, NC 27710

POLLACK, ARI, MD, MS *
ASSOCIATE PROFESSOR, ADJUNCT ASSOCIATE
PROFESSOR INFORMATION SCHOOL,
DIVISION OF NEPHROLOGY
DEPARTMENT OF PEDIATRICS
DEPARTMENT OF BIOMEDICAL INFORMATICS AND MEDICAL
EDUCATION, UNIVERSITY OF WASHINGTON
SEATTLE, WA 98195-9472

PRESCOTT, HALLIE CHRISTINE, MD, MS
ASSOCIATE PROFESSOR
DEPARTMENT OF INTERNAL MEDICINE
UNIVERSITY OF MICHIGAN
ANN ARBOR, MI 48109

RAMOS, SILVIA RAQUEL, PHD, MBA *
ASSOCIATE PROFESSOR OF NURSING AND PUBLIC HEALTH
SCHOOL OF NURSING
DEPARTMENT OF SOCIAL AND BEHAVIORAL SCIENCES
SCHOOL OF PUBLIC HEALTH
YALE UNIVERSITY
NEW HAVEN, CT 06520

REHG, JAMES M, PHD
PROFESSOR
CENTER FOR BEHAVIORAL IMAGING
SCHOOL OF INTERACTIVE COMPUTING
GEORGIA INSTITUTE OF TECHNOLOGY
ATLANTA, GA 30332

RIOS, ANTHONY, PHD *
ASSISTANT PROFESSOR
DEPARTMENT OF INFORMATION SYSTEMS
AND CYBER SECURITY
CARLOS ALVAREZ COLLEGE OF BUSINESS
UNIVERSITY OF TEXAS SAN ANTONIO
SAN ANTONIO, TX 78249

SEO, NA JIN, PHD
PROFESSOR
DEPARTMENTS OF HEALTH PROFESSIONS AND
HEALTH SCIENCES AND RESEARCH
MEDICAL UNIVERSITY OF SOUTH CAROLINA
CHARLESTON, SC 29425

SHYU, CHI-REN, PHD *
DIRECTOR, INSTITUTE FOR DATA SCIENCE AND INFORMATICS PAUL K. AND DIANNE SHUMAKER PROFESSOR
COLLEGE OF ENGINEERING
UNIVERSITY OF MISSOURI
COLUMBIA, MO 65211

SONG, XUBO, PHD *
PROFESSOR
DEPARTMENT OF COMPUTER SCIENCE AND ELECTRICAL ENGINEERING
SCHOOL OF MEDICINE
OREGON HEALTH AND SCIENCE UNIVERSITY
PORTLAND, OR 97239

SPIEGEL, BRENNAN, MD, MPH *
PROFESSOR OF MEDICINE AND PUBLIC HEALTH
DIRECTOR OF HEALTH SERVICES RESEARCH
CEDARS-SINAI CEDARS-SINAI SITE DIRECTOR
CLINICAL AND TRANSLATIONAL SCIENCE INSTITUTE
LOS ANGELES, CA 90048

STAVRAKIS, STAVROS, PHD, MD *
ASSOCIATE PROFESSOR OF MEDICINE
DIRECTOR, CARDIOVASCULAR RESEARCH
UNIVERSITY OF OKLAHOMA HEALTH SCIENCES CENTER
OKLAHOMA CITY, OK 73104

SWARD, KATHERINE ANN, PHD, RN
PROFESSOR
DEPARTMENT OF BIOMEDICAL INFORMATICS
COLLEGE OF NURSING
UNIVERSITY OF UTAH
SALT LAKE CITY, UT 84112

TANDON, ANIMESH, MD, MS *
DIRECTOR OF CARDIOVASCULAR INNOVATION
CLINICAL INNOVATION LEAD, PEDIATRIC INSTITUTE
CLEVELAND CLINIC
CLEVELAND, OH 44195

TOPAZ, MAXIM, PHD, RN
ASSOCIATE PROFESSOR
SCHOOL OF NURSING
COLUMBIA UNIVERSITY
NEW YORK, NY 10032

WADE, ERIC, PHD
ASSOCIATE PROFESSOR
DEPARTMENT OF MECHANICAL, AEROSPACE AND BIOMEDICAL ENGINEERING
UNIVERSITY OF TENNESSEE, KNOXVILLE
KNOXVILLE, TN 37996

WEI, JEANNE Y, PHD, MD *
EXECUTIVE DIRECTOR, REYNOLDS INSTITUTE ON AGING
PROFESSOR AND CHAIRPERSON
REYNOLDS DEPARTMENT OF GERIATRICS
COLLEGE OF MEDICINE
UNIVERSITY OF ARKANSAS FOR MEDICAL SCIENCES
LITTLE ROCK, AR 72205

MAIL REVIEWER(S)

LAKSHMINARAYAN, KAMAKSHI, PHD, MBBS, MS
PROFESSOR
DEPARTMENT OF NEUROLOGY, MEDICAL SCHOOL
SCHOOL OF PUBLIC HEALTH
UNIVERSITY OF MINNESOTA
MINNEAPOLIS, MN 55454

SCIENTIFIC REVIEW OFFICER

HEWETT, PAUL, PHD
SCIENTIFIC REVIEW OFFICER
CENTER FOR SCIENTIFIC REVIEW
NATIONAL INSTITUTE OF HEALTH
BETHESDA, MD 20892

EXTRAMURAL SUPPORT ASSISTANT

NJOKU, PHILIP C
EXTRAMURAL SUPPORT ASSISTANT
DIVISION OF AIDS, BEHAVORIAL, POPULATION SCIENCES
NATIONAL INSTITUTES OF HEALTH
BETHESDA, MD 20892

* Temporary Member. For grant applications, temporary members may participate in the entire meeting or may review only selected applications as needed.

Consultants are required to absent themselves from the room during the review of any application if their presence would constitute or appear to constitute a conflict of interest.
